# Heme Oxygenase-1 (HO-1) Expression in Prostate Cancer Cells Modulates the Oxidative Response in Bone Cells

**DOI:** 10.1371/journal.pone.0080315

**Published:** 2013-11-04

**Authors:** Mercedes Ferrando, Xinhai Wan, Roberto Meiss, Jun Yang, Adriana De Siervi, Nora Navone, Elba Vazquez

**Affiliations:** 1 Department of Biological Chemistry, School of Sciences, University of Buenos Aires, IQUIBICEN-CONICET, Buenos Aires, Argentina; 2 Department of Genitourinary Medical Oncology, the University of Texas, M. D. Anderson Cancer Center, Houston, Texas, United States of America; 3 Department of Pathology, Institute of Oncological Studies, National Academy of Medicine, Buenos Aires, Argentina; Innsbruck Medical University, Austria

## Abstract

Prostate cancer (PCa) is a leading cause of death among males. It is currently estimated that inflammatory responses are linked to 15-20% of all deaths from cancer worldwide. PCa is dominated by complications arising from metastasis to the bone where the tumor cells interact with the bone microenvironment impairing the balance between bone formation and degradation. However, the molecular nature of this interaction is not completely understood. Heme oxygenase-1 (HO-1) counteracts oxidative damage and inflammation. Previous studies from our laboratory showed that HO-1 is implicated in PCa, demonstrating that endogenous HO-1 inhibits bone derived-prostate cancer cells proliferation, invasion and migration and decreases tumor growth and angiogenesis *in vivo*. The aim of this work was to analyze the impact of HO-1 modulated PCa cells on osteoblasts proliferation *in vitro* and on bone remodeling *in vivo*. Using a co-culture system of PC3 cells with primary mice osteoblasts (PMOs), we demonstrated that HO-1 pharmacological induction (hemin treatment) abrogated the diminution of PMOs proliferation induced by PCa cells and decreased the expression of osteoclast-modulating factors in osteoblasts. No changes were detected in the expression of genes involved in osteoblasts differentiation. However, co-culture of hemin pre-treated PC3 cells (PC3 Hem) with PMOs provoked an oxidative status and activated FoxO signaling in osteoblasts. The percentage of active osteoblasts positive for HO-1 increased in calvarias explants co-cultured with PC3 Hem cells. Nuclear HO-1 expression was detected in tumors generated by in vivo bone injection of HO-1 stable transfected PC3 (PC3HO-1) cells in the femur of *SCID* mice. These results suggest that HO-1 has the potential to modify the bone microenvironment impacting on PCa bone metastasis.

## Introduction

Bone metastases dominate the clinical picture of advanced prostate cancer (PCa) and are responsible for most of the mortality and morbidity of the disease. Metastases to bone may have either an osteolytic (bone resorbing) or an osteoblastic (bone producing) phenotype. PCa characteristically produces osteoblastic lesions in bone, although an osteolytic component is always present. 

It has been suggested that inflammation increases prostate tumorigenesis [1]. Cytokines, chemokines and matrix metalloproteinases are part of a proinflammatory network that contributes to malignant progression [2]. Indeed, proinflammatory factors secreted by PCa and bone cells and the subsequent released of factors from the organic matrix in the bone, mediate a paracrine/autocrine interaction between PCa cells, osteoblasts and osteoclasts, which ultimately determine the bone phenotype and progression of PCa [3,4]. 

Oxidative stress is a natural consequence of inflammatory processes and acts as a modulator of the function of mineralized tissues [5]. It affects bone formation by inhibiting the differentiation of osteoblasts and promoting apoptosis [6]. These effects are mediated in part by reactive oxygen species (ROS) generated in the context of oxidative stress. Cells counteract the adverse effects of ROS by activating various defense mechanisms, including induction of free radical scavenging enzymes such as manganese superoxide dismutase (MnSOD), catalase, and also DNA repair genes. This response requires the activation of a family of ubiquitous factors known as forkhead transcription box O (FoxO) [7], which through interaction with β-catenin reduces oxidative stress and promotes osteoblasts survival [6].

Heme oxygenase 1 (HO-1), the rate-limiting enzyme in heme degradation, was demonstrated to confer cytoprotection against oxidative stress and inflammation in several animal models [8]. Previous reports from our laboratory documented for the first time the nuclear expression of HO-1 in human primary prostate carcinomas [9]. We also documented that HO-1 inhibits cell proliferation, migration, and invasion *in vitro* and impairs PCa tumor growth *in vivo* [10]. In addition, we previously established a key role for HO-1 as a modulator of the angiogenic switch in prostate carcinogenesis [11]. Moreover, we showed evidence that the anti-angiogenic function of HO-1 was mediated by repression of the nuclear factor kappa-light-chain-enhancer of activated B cells (NF-κB) signaling pathway [11]. Recently, we reported a novel function for HO-1 in PCa. We demonstrated that HO-1 down-modulates AR transcriptional activity by interfering with STAT3 signaling and provided evidence of its role beyond heme degradation [12].

The studies presented here were performed using a bone derived osteolytic PCa cell line (PC3) that has been shown to inhibit osteoblast proliferation *in vitro* in a co-culture system with primary mouse osteoblasts (PMOs) [13]. In this paper we report data that support a novel function for HO-1 in the interaction between PCa cells and bone. We found that HO-1 induction in PC3 cells restored the proliferation of osteoblasts, which was inhibited during co-culture with parental PC3 cells. Our studies suggest that these effects are mediated by the activation of FoxO signaling in osteoblasts. PC3 cells overexpressing HO-1 (PC3HO-1) growing in the bone of mice, produced tumors with nuclear localization of HO-1 as detected by immunohistochemistry. These data indicate that HO-1 plays a role in the progression of PCa in bone. A better understanding of the molecular mechanisms underlying the interplay between PCa cells and bone microenvironment may help to identify novel targets for pharmacological intervention in this disease.

## Materials and Methods

### Cell Cultures and Antibodies

The prostate cancer cell line PC3 was obtained from the American Type Culture Collection (Manassas, VA, USA) and routinely cultured in RPMI 1640 (Invitrogen, CA, USA) supplemented with 10% fetal bovine serum (FBS). PC3 stable transfected cells (PC3HO-1 and PC3βgal) were generated as described previously [10]. The mouse myoblast cell line C2C12 was obtained from the American Type Culture Collection (Manassas, VA, USA) and was cultured in DMEM low-glucose (Invitrogen, CA, USA) with 10% FBS. Primary cultures of mice osteoblasts (PMOs) were established by a procedure published previously [13] and were obtained from the calvaria of newborn CD1 mice sacrificed 4 days after birth. Isolated cells were plated in α-MEM (Invitrogen, CA, USA) plus 10% FBS for 48 h. The PMOs were then subsequently trypsinized and replated in culture dishes to perform experiments. Antibodies: anti–HO-1 was from Stressgen Biotechnologies, Corp. (San Diego, CA, USA), anti-β-Catenin was from BD Transduction Laboratories™ (CA, USA), anti–β-actin was from Cell Signaling Technology (Beverly, MA, USA), anti-cyclin B1, anti-cyclin A, anti-cyclin D1, anti-p21, anti-β-tubulin, anti-mouse and anti-rabbit secondary antibodies were from Santa Cruz Biotechnology Inc. (CA, USA) and Alexa Fluor® 594-conjugated secondary antibody from Invitrogen (CA, USA).

### Hemin pre-treatment of PCa cells and co-culture system

An *in vitro* bicompartment culture system was used as a model of bone metastases from PCa as previously described slightly modified [13]. In brief, on day 0 PC3 cells were seeded (10,500 cell/cm^2^) in cell-culture inserts (0.4-mm pore; Falcon/ Becton Dickinson Labware, Franklin Lakes, NJ, USA) and on day 1 they were treated with hemin (80 μM, Sigma-Aldrich, St Louis, MO, USA), a potent inducer of HO-1 (PC3 Hem). Controls received fresh medium. The PMOs were also seeded on day 1 in tissue culture plates (7,300 cell/cm^2^). On day 2, the inserts containing the PC3 cells (pre-treated or not with hemin) were extensively washed with PBS. Then, the inserts were placed into tissue-culture plates containing the PMOs so that the two different cell types shared the culture medium but were not in physical contact. Co-culturing of PC3 cells with PMOs was performed with α-MEM plus 2% FBS for 24 h. On day 3, the cells were collected and different parameters were analyzed. As control, each cell type (PC3 cells pre-treated or not with hemin and PMOs) was grown alone. Co-cultures of C2C12 cells and PC3 were done in the same conditions like PMOs and PC3 cells. Cultures were done in triplicate and each experiment was assayed three times.

### Mitogenic assay

Proliferation and DNA synthesis were assessed by adding [^3^H]-thymidine (Amershan Biosciencies) during the final 3 h of co-culture and incorporation was measured as described elsewhere [14].

### RNA isolation and RT-qPCR (reverse transcription quantitative PCR)

Total RNA was isolated with the RNeasy Mini Kit (Qiagen). cDNAs were synthesized with RevertAid RT (Fermentas) and amplified by real-time PCR amplification with Taq DNA Polimerase (Fermentas). Primers were designed using Beacon Designer 5 and tested with UCSC Genome Browser Home URL. The PCR was performed in a DNA Engine Opticon (MJ Research). Human primer sequences were given as follows: ACTB 5´-AAGATCATTGCTCCTCCTGAGC-3´ and 5´-CATACTCCTGCTTGCTGATCCA-3´; HO-1 5´-GAGTGTAAGGACCCATCGGA-3´ and 5´-GCCAGCAACAAAGTGCAAG-3´; PTHrP 5´-GTCTCAGCCGCCGCCTCAA-3´ and 5´-GGAAGAATCGTCGCCGTAAA-3´; uPA 5´-GAGATCACTGGCTTTGGAAAA-3´ and 5´-CCAGCTCACAATTCCAGTCA-3´; TGF-β1 5´-TACCTGAACCCGTGTTGCTC-3´ and 5´-GCGAAAGCCCTCAATTTCCC-3´. Mouse primer sequences were given as follows: ACTB 5´-CCGCACGACAACCGCACCAT-3´ and 5´-CGGTTGGCCTTAGGGTTCAGGGGGG-3´; Runx-2 5´-CCGCACGACAACCGCACCAT-3´ and 5´-AGGCATTTCGGAGCTCGGCG-3´; ALP 5´-AACCCAGACACAAGCATTCC-3´ and 5´-GAGACATTTTCCCGTTCACC-3´; Col1a1 5´-CATGTTCAGCTTTGTGGACCT-3´ and 5´-GCAGCTGACTTCAGGGATGT-3´; Col1a2 5´-GCAGGTTCACCTACTCTGTCCT-3´ and 5´-CTTGCCCCATTCATTTGTCT-3´; OCN 5´-GCAGCTTGGTGCACACCTAG-3´ and 5´-GGAGCTGCTGTGACATCCATAC-3´; RANKL 5´-TGATTCATGTAGGAGAATTAAACAGG-3´ and 5´-GATGTGCTGTGATCCAACGA-3´; OPG 5´-GAAGGGCGCTACCTTGAGAT-3’ and 5´-GCAAACTGTATTTCGCTCTGG-3´; CSF-1 5´-CAACAGCTTTGCTAAGTGCTCTA-3´ and 5´-CACTGCTAGGGGTGGCTTTA-3´; OPN 5´-CTTTCACTCCAATCGTCCCTA-3´ and 5´-GCTCTCTTTGGAATGCTCAAGT-3´; CCL2 5´-TGCTACTCATTAACCAGCAAGAT-3´ and 5´-TGCTTGAGGTGGTTGTGGAA-3´; IL-6 5´-CTGCAAGAGACTTCCATCCAGTT-3´ and 5´-GAAGTAGGGAAGGCCGTGG-3´; MnSOD 5´-CCACACATTAACGCGCAGATC-3´ and 5´-TAACATCTCCCTT GGCCAGAGC-3´; catalase 5´-TTGCTGAAGTTGAACAGATGG-3´ and 5´-ATCACGCTGGTAGTTGGC-3´.

### Western blot analysis

For western blotting, 50 µg of protein was separated on 12% Tris-glycine polyacrylamide gels and transferred to nitrocellulose membranes (BIORAD POWERPAC Basic). The specific proteins were detected by chemiluminescence (Amersham) as previously described [10].

### Plasmids, transfections and luciferase reporter assay

A TOP-flash reporter gene construct containing four consensus TCF binding sites, a minimal Fos binding site and a luciferase reporter was used [15]. A FOP-flash construct with a mutated TCF binding site was used as a negative control. A reporter plasmid containing 6 copies of daf-16 family protein binding element (FoxO-luc) in the pGL3-basic firefly luciferase vector with a minimal TATA box [16] was kindly provided by B. Burgering, University Medical Center, Utrecht, Netherlands. Luciferase reporter constructs were introduced into C2C12 cells by transient transfection using 8 µg of PEI and 4 µg of plasmid. After 6 h, the medium was removed and the C2C12 were co-cultured with PC3 cells pre-treated or not with hemin or grown alone for 24 h. C2C12 cells were then harvested and lysed with 40 μl of Steady Glo Luciferase System (Promega, Madison, WI, USA). Luciferase activity was measured in a luminometer (Glomax Multi Detection System; Promega). As positive control of TCF binding sites activation, PMOs were treated with LiCl 10 mM for 24 h and of FoxO-luc activation, cells were exposed to H_2_O_2_ 100µM for 24 h. Each transfection was done in triplicate and each experiment was repeated at least three times. Data were normalized to total protein determined by the Bradford assay.

### Assessment of reactive oxygen species by flow cytometry

After the co-culture, the PMOs were washed with PBS and incubated with 10 µM 2´,7´- Dichlorofluorescein diacetate (CM2-DCFHDA; Invitrogen-Molecular Probes^TM^) for 1 h at 37°C. The cells were tripsined and resuspended with PBS. The levels of H_2_DCFDA were measured by flow citometry in the FITC channel.

### Cell cycle

After the co-culture, PMOs were fixed and stained with propidium iodide (PI), and analyzed by fluorescence-activated cell sorting (FACS) as previously described [17].

### Immunofluorescence

PMOs were co-cultured as described previously but seeded on coverslips. The immunofluorescence analyses were performed as previously described [12]. Wide field microscopy was carried out using an Olympus IX71 microscope with a water immersion objective (UPLSAPO 60XW 1,2 AN, Olympus). Images were taken with the camera Hamammatsu Orca-ER using the software Andor IQ and were processed for presentation with ImageJ (http://rsb.info.nih.gov, National Institutes of Health).

### Immunohistochemical analyses

Immunohistochemical technique was performed as previously described [9,10]. For quantitative analysis, the total number of osteoblasts in at least 6 fields was counted and the percentage of osteoblasts with positive immunoreactivity for the studied gene was calculated. 

### Organ culture

Calvaria from 4-day-old CD1 mouse pups were excise, cut in half and placed in BGJ medium (Sigma Aldrich) containing 0.1% bovine serum albumin on a cell culture insert that suspends the bone organ between atmosphere and medium for optimal CO_2_ exchange. Half of each calvaria was co-cultured with PC3 cells pre-treated with hemin and the other half with PC3 untreated cells. Other calvarias were placed in BGJ medium containing 0.1% bovine serum albumin and used as controls. Other halves treated with insulin 20 µg/ml were used as positive controls of bone formation. The corresponding PC3 culture and the medium were changed every 2 days and the experiment was terminated at the end of 7 days. At that time, the calvaria halves were fixed, decalcified, paraffin-embedded, sectioned, stained with hematoxylin and eosin or immunohistochemical stainned and analyzed as previously described [13].

### *In vivo* prostate cancer intrabone model

All animal experiments were conducted in accordance with accepted standards of animal care and were approved by the Institutional Animal Care and Use

Committee of the University of Texas MD Anderson Cancer Center. PC3HO-1 or PC3βgal cells were injected into the femurs of male SCID mice (Charles River Laboratories, Wilmington, MA, USA) as previously described [13]. 3 × 10^5^ PCa cells per mouse were injected with a 28-gauge needle into the distal ends of the right femurs of 6- to 8-week-old intact male SCID mice (n=7 per group) according to procedures described elsewhere. Bone formation was assessed by X-ray analysis. At the end of the experiment, mice were euthanized by cervical dislocation after anesthesia with isoflurane, (delivered by the open-*drop method*) after which their hind legs were removed and the muscle tissues dissected from the bones of both the injected and control hind limbs of mice bearing the femoral xenografts. The dissected bones were then processed for histologic. 

### Statistical analysis

All results are given as mean ± SD of 3 separate independent experiments unless stated otherwise. Student’s t test was used to ascertain statistical significance with a threshold of *P*<0.05 (*), *P*<0.01(**) and *P*<0.001 (***).

## Results

### HO-1 expression in PC3 cells impairs growth inhibitory effects of tumor cells on osteoblasts proliferation

**Figure 1:**
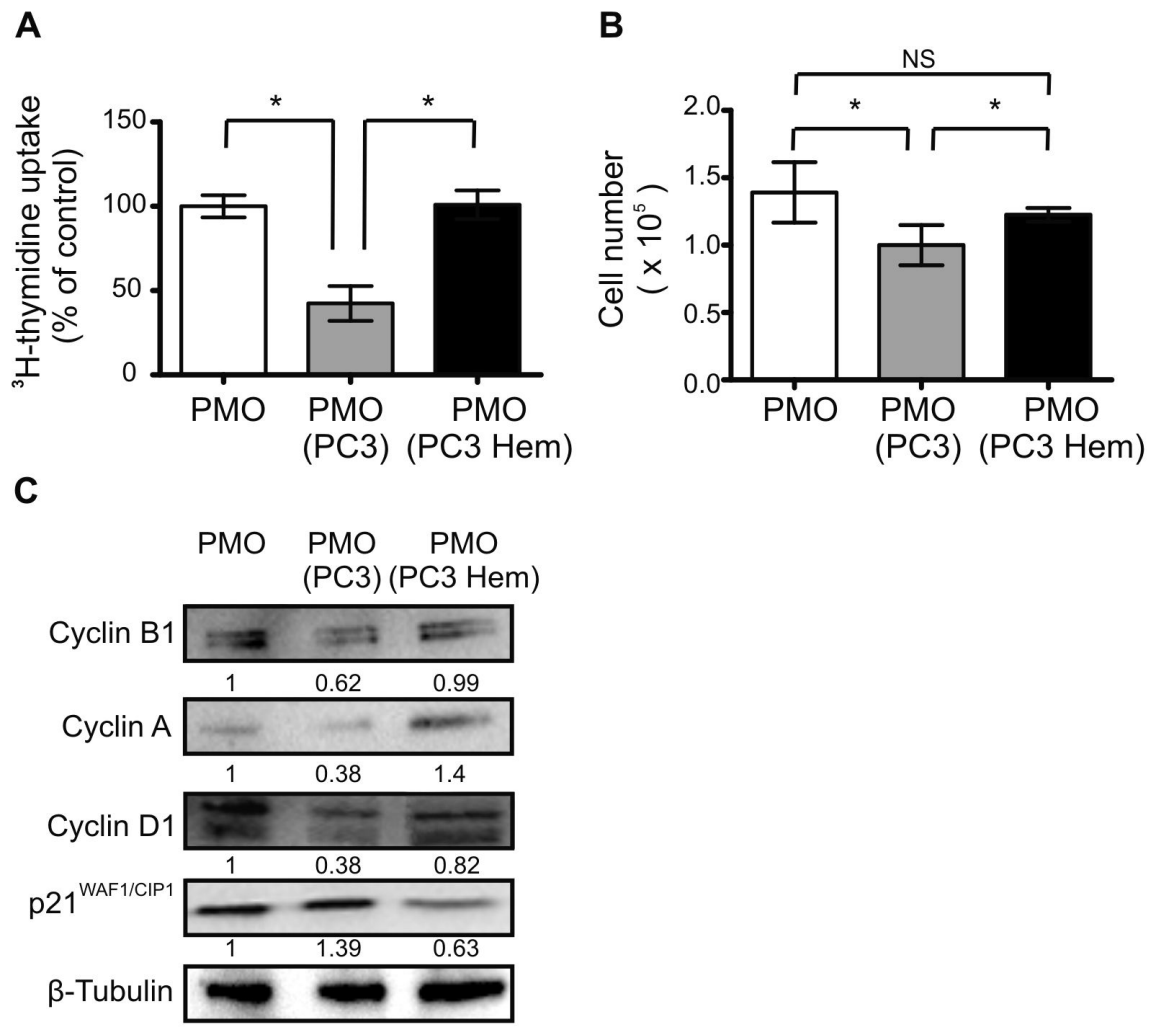
Co-culture with HO-1-induced PC3 cells restored PMOs proliferation. Experiments were done in PMOs grown alone (PMO), co-cultured with PC3 (PMO PC3) or with PC3 pre-treated with hemin (PMO PC3 Hem). **A**: Cell proliferation was measured by ^3^H-thymidine incorporation in PMOs after 24h culture. Results are expressed as a percentage of the PMOs monoculture set at 100%. **B**: PMOs cell number. **C**: Western blot analysis was carried out by using anti-cyclin B1, A, and D1 and anti–p21^WAF1/CIP1^ antibodies. The numbers under the bands indicates the quantitation normalized to β-Tubulin and PMOs alone. One representative from at least three independent experiments is shown (Significant difference, * *P*<0.05; No significant difference, NS).

*In vivo*, PC3 cells predominantly produce bone-resorbing effects leading to osteolytic bone lesions [18]. *In vitro*, it was well documented that when PMOs were co-cultured with PC3 cells the number of PMOs decreased significantly [13]. In order to analyze the effect of HO-1 on the interaction between PCa cells and osteoblasts, HO-1 expression in PC3 cells was induced by pre-treatment with hemin (PC3 Hem) (80 μM, 24 h). PC3 Hem and control cells were then co-cultured with PMOs during 24 h. We confirmed the inhibitory effect of PC3 cells on osteoblasts proliferation as assessed by ^3^H-thymidine incorporation and cell number (57% and 28%, *P*<0.05; respectively) (Figure 1 A&B). Noticeably, the co-culture of PMOs with PC3 Hem restored osteoblasts proliferation at levels found in PMOs growing alone (Figure 1 A&B), further emphasizing the pro-homeostatic role of HO-1. The induction of HO-1 expression in PC3 cells was confirmed at mRNA and protein levels (Figure 1).

The analyses of proteins involved in cell cycle regulation by immunoblotting in PMOs after co-culture with PC3 Hem showed an increase in the levels of cyclin A, cyclin B and cyclin D1, with a concomitant decrease in p21^WAF1/CIP1^ expression as compared to PMOs grown in co-culture with PC3 control cells (Figure 1C). Furthermore, flow cytometric analysis of PMOs after co-culture with PC3 Hem demonstrated significant increased on G2/M accumulation as compared to PMOs co-culture with PC3 control cells (*P*<0.05) (data not shown). All these data suggest that HO-1 expression in PCa cells induces cell cycle progression in PMOs, restoring the growth rate of PMOs alone. It is worth to remark that the number of PC3 cells was not modified in the different co-culture conditions assayed (data not shown).

### HO-1 expression in PC3 cells does not alter the effects of tumor cells on osteoblasts differentiation

**Figure 2:**
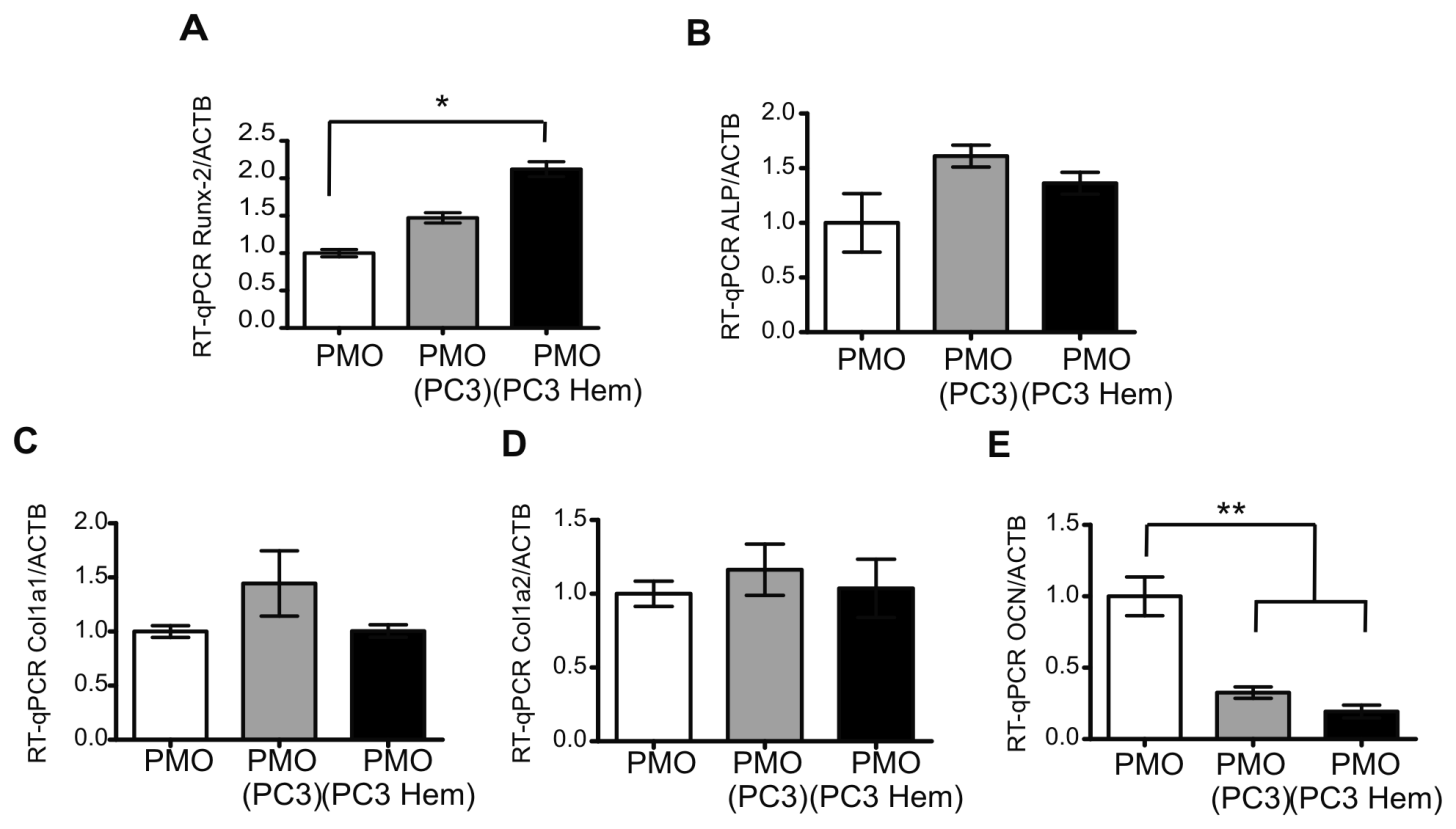
Co-culture with HO-1-induced PC3 cells did not affect osteoblasts differentiation. Experiments were done in PMOs grown alone (PMO), co-cultured with PC3 (PMO PC3) or with PC3 pre-treated with hemin (PMO PC3 Hem). Total RNA was extracted and Runx-2 (**A**), ALP (**B**), collagen type I (C), collagen type II (D) and OCN (**E**) mRNA levels were analyzed by RT-qPCR. Data were normalized to β-actin and were expressed as fold induction respect to PMOs. One representative from at least three independent experiments is shown (Significant difference, * *P*<0.05; ** *P*<0.01).

Transcript levels of various osteoblasts specific genes were assessed by RT-qPCR. The expression of the osteoblasts specific transcription factor Runx-2, of an early differentiation marker (alkaline phosphatase, ALP), of late differentiation markers involved in collagenous matrix deposition (collagen type I and collagen type II) and non-collagenous matrix deposition (osteocalcin, OCN) was analyzed. We found that Runx-2 expression was increased in PMOs co-cultured with PC3 Hem (130%, *P*<0.05) (Figure 2 A) but no differences were detected in the levels of ALP, collagen type I and II in PMOs growing alone or in co-culture with PC3 Hem or PC3 control cells (Figure 2 B-D). OCN levels were significantly decreased in PMOs co-cultured with PC3 Hem or PC3 controls (81% and 68%, *P*<0.01; respectively) (Figure 2 E). In agreement with previous reports [13,19,20], PMOs co-cultured with PC3 cells exhibited a decrease in calcified matrix formation as assessed by von Kossa staining compared with PMOs growing alone (Figure 2). No alteration was detected on calcified matrix when PMOs were co-cultured with PC3 Hem (Figure 2). These results demonstrate that PC3-secreted factors, reported to be responsible of the inhibition of osteoblasts differentiation [13], are not modified when HO-1 expression is induced in tumor cells.

### HO-1 expression in PC3 cells decreased the levels of osteoclast-modulating factors in osteoblasts

**Figure 3:**
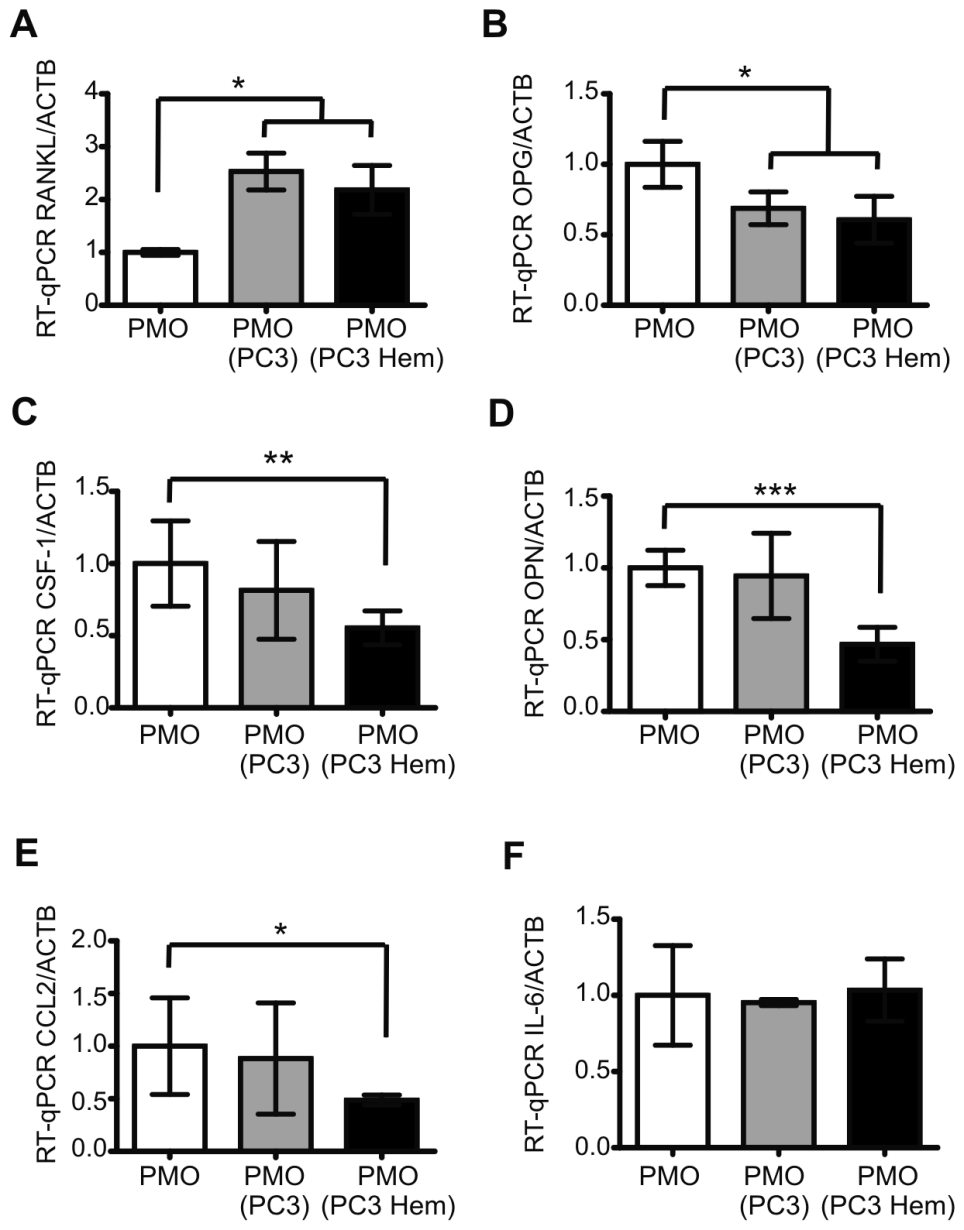
Co-culture with HO-1-induced PC3 cells decreased the expression of osteoclast-modulating factors in osteoblasts. Experiments were done in PMOs grown alone (PMO), co-cultured with PC3 (PMO PC3) or with PC3 pre-treated with hemin (PMO PC3 Hem). Total RNA was extracted and RANKL (**A**), OPG (**B**), CSF-1 (**C**), OPN (**D**), CCL2 (**E**) and IL-6 (**F**) mRNA levels were analyzed by RT-qPCR. Data were normalized to β-actin and were expressed as fold induction respect to PMOs. One representative from at least three independent experiments is shown (Significant difference, * *P*<0.05; ** *P*<0.01; *** *P*<0.001).

Receptor activator of nuclear factor kappa-B ligand (RANKL) and Osteoprotegerin (OPG) are produced by osteoblastic/stromal cells. RANKL has been shown to activate mature osteoclasts and mediate osteoclastogenesis. OPG acts as a decoy receptor for RANKL [21]. The co-culture of PMOs and PC3 Hem cells produced an increase in RANKL and a decrease in OPG transcript levels in osteoblasts; the same result was observed after co-culture of PMOs with PC3 control cells (Figure 3 A&B).

Osteoblasts also produce cytokines and other growth factors that promote osteoclasts development and activation, such as colony stimulating factor-1 (Csf-1), osteopontin (OPN), chemokine (C-C motif) ligand 2 (CCL2) and interleukin-6 (IL-6). We found that PMOs co-cultured with PC3 Hem expressed significant lower transcript levels of Csf-1, OPN and CCL2 as compared to PMOs grown alone (52%, *P*<0.01; 53%, *P*<0.001 and 52%, *P*<0.05; respectively). No differences were detected in IL-6 levels (Figure 3 C-F).

**Figure 4:**
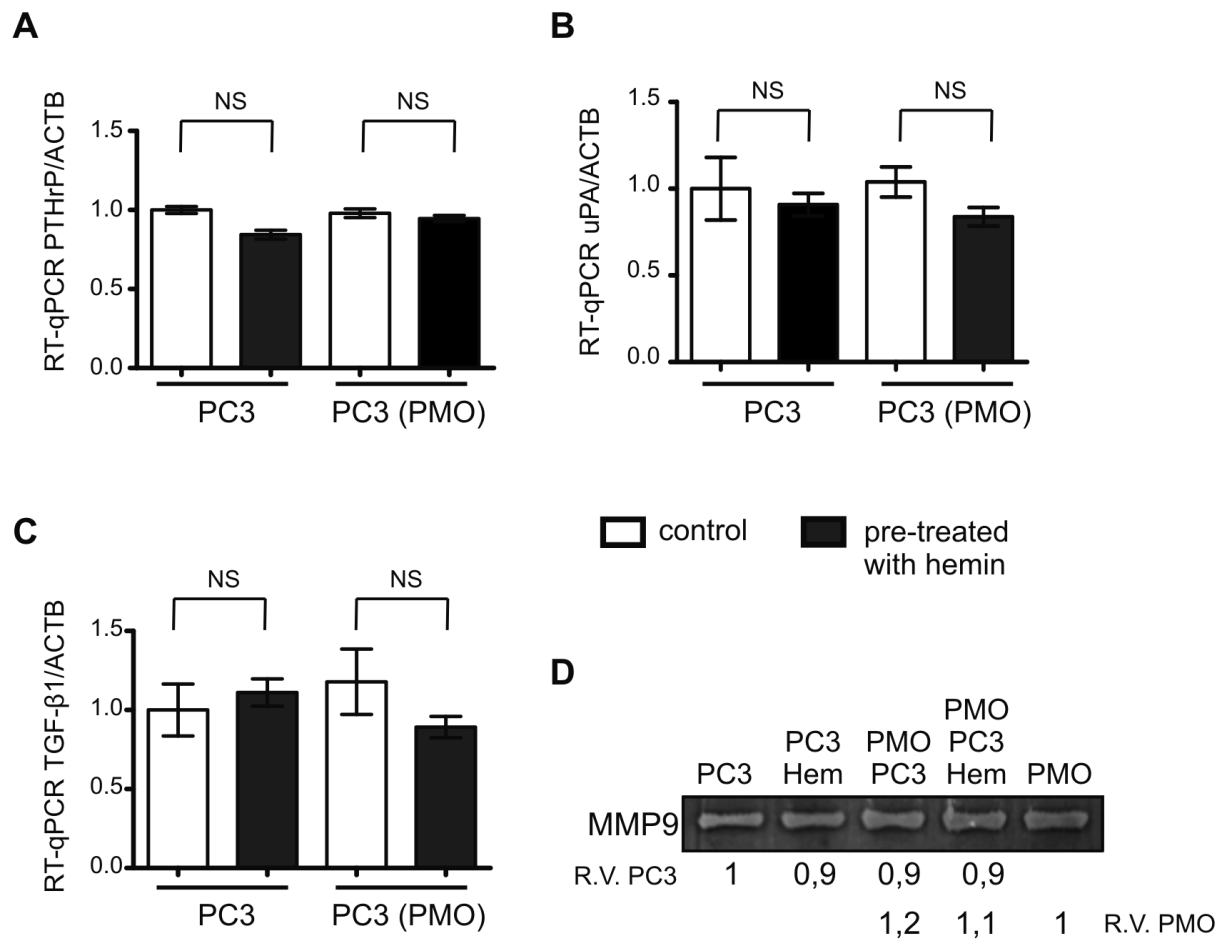
The expression of genes implicated in osteoclasts modulation is not altered in PC3 cells by co-culturing with osteoblasts. PC3 cells were pre-treated with hemin (80 μM, 24 h, black columns) or not (control, white columns) and co-cultured or not with PMOs. Total RNA was extracted and PTHrP (**A**), uPA (**B**) and TGF-β1 (**C**) mRNA levels were analyzed by RT-qPCR. Data were normalized to β-actin and were expressed as fold induction respect to PC3. Gelatin zymography was done to assess MMP9 activity on conditioned media from PC3 grown alone (PC3), pre-treated with hemin (PC3 Hem) and from PMOs grown alone (PMO) and co-cultured with PC3 pre-treated or not with hemin (PMO PC3 Hem and PMO PC3, respectively). Clear bands were quantified using ImageJ 1.37.v software (NIH) and normalized to total protein. One representative from at least three independent experiments is shown (NS: No significant difference; R.V.: relative values).

PCa cells also express genes indirectly involved in osteoclasts modulation. Therefore, we decided to investigate the expression in PC3 cells of parathyroid hormone-related peptide (PTHrP), urokinase-type plasminogen activator (uPA) and transforming growth factor beta 1 (TGF-β1) by RT-qPCR and the activity of matrix metalloprotease 9 (MMP9) by zymography. No differences were observed in the transcripts levels of the selected genes or in the activity of MMP9 (Figure 4), indicating that these genes implicated in osteoclasts modulation are not altered in PC3 cells under the experimental conditions either pre-treated or not with hemin growing alone or in co-culture.

### Onset of osteoblasts oxidative status by co-culture with hemin pre-treated PC3 cells

**Figure 5:**
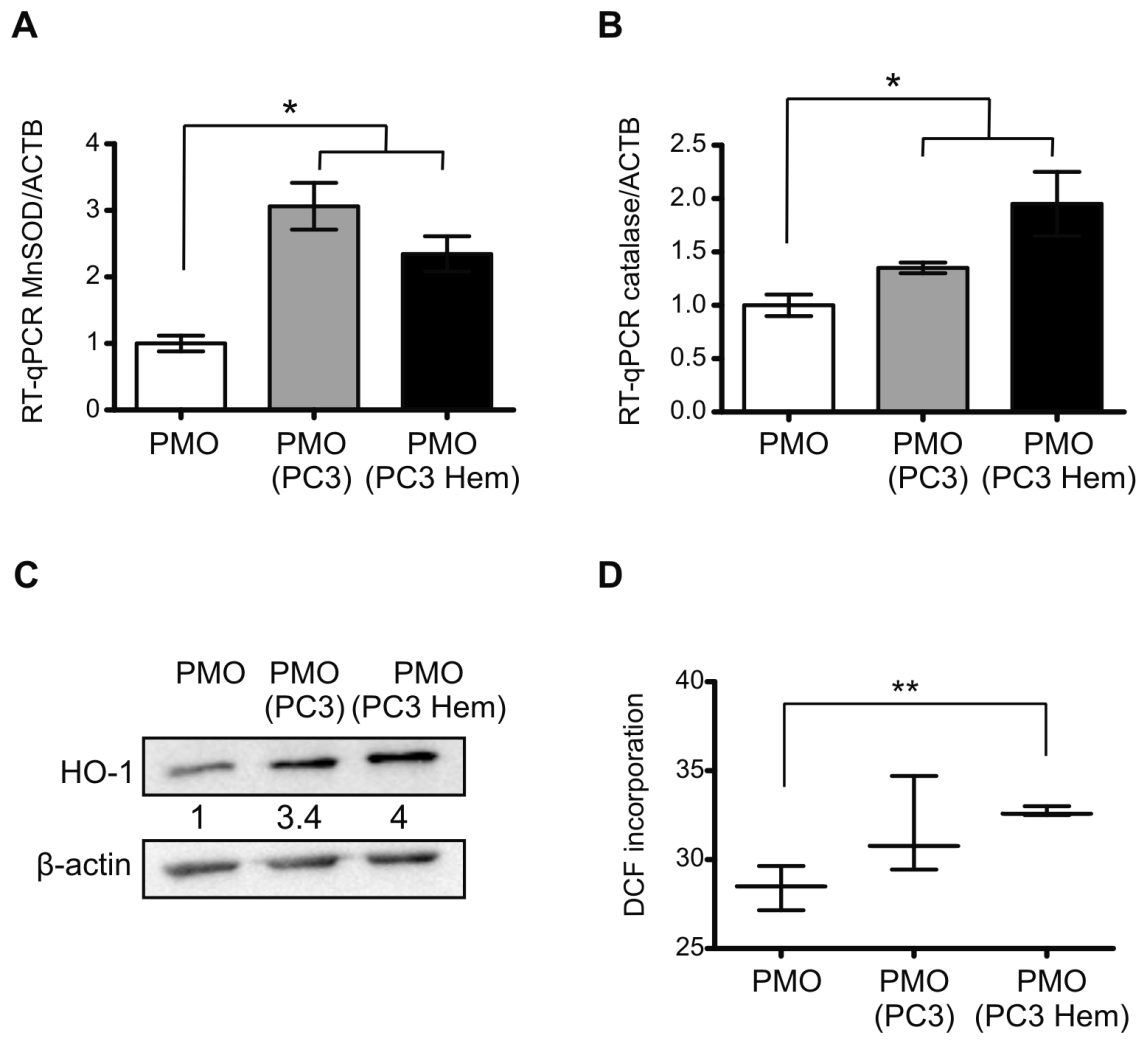
Co-culture with HO-1-induced PC3 cells induced oxidative stress markers in osteoblasts. Experiments were done in PMOs grown alone (PMO), co-cultured with PC3 (PMO PC3) or with PC3 pre-treated with hemin (PMO PC3 Hem). Total RNA was extracted and MnSOD (**A**) and catalase (**B**) mRNA levels were analyzed by RT-qPCR. Data were normalized to β-actin and were expressed as fold induction respect to PMOs. HO-1 protein levels (**C**) were determined by Western blot analysis. The numbers under the bands indicates the quantitation normalized to β-actin and PMOs alone. ROS levels (**D**) were determined in PMOs incubated with CM2-DCFHDA and measured by flow citometry in the FITC channel (Significant difference, * *P*<0.05; ** *P*<0.01).

Previous reports indicate that oxidative stress leads to decreased osteoblasts number and bone formation rate [22]. In order to investigate whether PCa cells induce oxidative stress in osteoblasts, we used our co-culture system to analyze the expression of antioxidant response genes (MnSOD, catalase and HO-1). The co-culture of PMOs with PC3 Hem or PC3 controls increased the transcript levels of MnSOD (134% and 206%, *P*<0.05; respectively) and catalase (95% and 35%, *P*<0.05; respectively) in PMOs (Figure 5 A&B). Interestingly, HO-1 protein expression in osteoblasts co-cultured with PC3 Hem or PC3 controls was strongly induced compared to PMOs growing alone (Figure 5 C). To determine the oxidative stress levels, the generation of ROS was measured by flow cytometry monitoring H_2_DCFDA oxidation. Significant increased levels of ROS were found in PMOs co-cultured with PC3 Hem compared to PMOs growing alone (15%, *P*<0.01) (Figure 5 D). Together these findings suggest that induced HO-1 expression in PC3 cells releases soluble factors leading to oxidative stress in PMOs. This in turn induces an antioxidant response probably favoring osteoblasts proliferation [6].

### HO-1 expression in PC3 cells activates FoxO signaling in C2C12 cells

**Figure 6:**
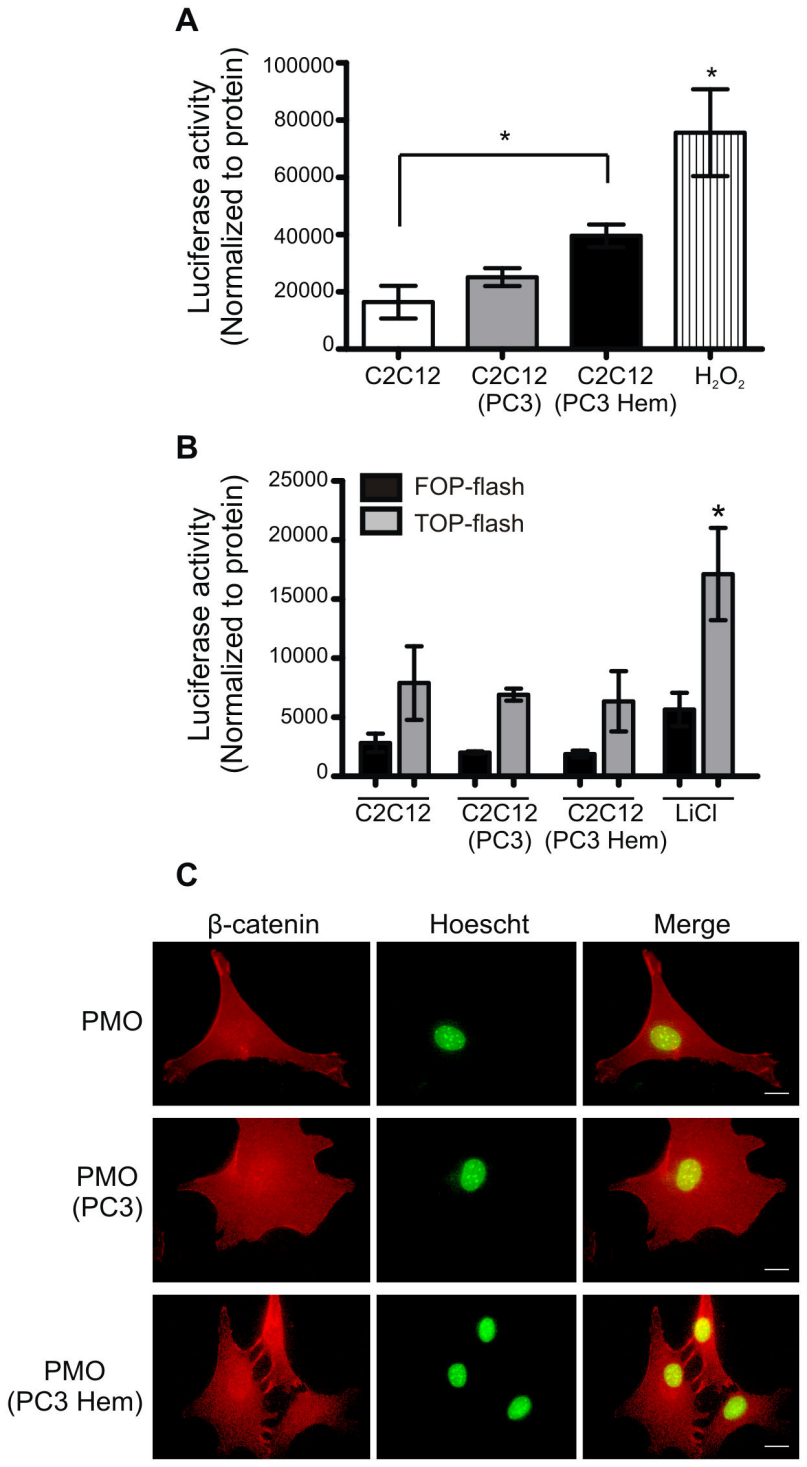
Co-culture with HO-1-induced PC3 cells did not change β-catenin localization but activated FoxO signaling in osteoblasts. **Upper panel**. Experiments were done in C2C12 grown alone (C2C12), co-cultured with PC3 (C2C12 PC3) or with PC3 pre-treated with hemin (C2C12 PC3 Hem). **A**: The cells were transfected with a FoxO reporter gene construct (FoxO-luc). H_2_O_2_ was used as a positive control. **B**: The cells were transfected with a TCF reporter gene construct (TOP-flash) or its negative control (FOP-flash). LiCl was a positive control. Six hours after transfection, the C2C12 cells were co-cultured and 24h after co-culturing the C2C12 cells were lysed and luciferase activity assay was done. Data were normalized to protein values. One representative from at least three independent experiments is shown (Significant difference, * *P*<0.05). **Lower panel**. **C**: β-catenin cellular distribution was visualized by immunofluorescence staining (red) of PMOs grown alone (PMO), co-cultured with PC3 (PMO PC3) or with PC3 pre-treated with hemin (PMO PC3 Hem). The nucleus was labelled using Hoescht stain (green). The Merge represents the overlapping images. A representative image for each group is shown. Scale bar: 30 μm.

In order to identify signaling pathways activated by oxidative stress in osteoblasts, we analyzed β-catenin/Foxo axis. Although canonical Wnt/β-catenin pathway plays a pivotal role in regulating osteoblast differentiation and bone formation, it is well known that soluble β-catenin interacts with FoxO transcription factors in response to oxidative stress [6]. To study this signaling pathway we used a reporter construct with six FoxO response elements. Due to the difficulty to transfect PMOs, we used an uncommitted mesenchymal cell line, C2C12, able to differentiate into the osteoblastic lineage [23]. The co-culture of C2C12 cells with PC3 Hem strongly stimulated the activity of the reporter gene (Figure 6 A). As positive control C2C12 cultures were exposed to H_2_O_2_ (100 μM) (Figure 6 A). The results outlined here suggest that soluble factors produced by PC3 Hem induce FoxO signaling in osteoblasts.

In the Wnt canonical pathway, β-catenin activation results in stabilization of the protein translocation into the nucleus, where it interacts with TCF (T-cell factor, HMG box) and activates transcription of target genes [24]. It has been suggested that Foxo divert β-catenin from the Wnt canonical pathway [6]. To assess whether Wnt canonical pathway in PMOs is altered after co-culture with PCa cells, we examined β-catenin subcellular localization and TCF activation in PMOs growing alone or in co-culture with PC3 Hem and PC3 control cells. The images showed in Figure 6 C demonstrate that β-catenin is located both in the nucleus and in the cytoplasm in all the analyzed conditions. To evaluate the activation of the β-catenin/TCF pathway we used a reporter gene. We co-cultured PC3 cells pre-treated or not with hemin with C2C12 cells that had been transfected with a TCF reporter gene construct (TOP-flash) [15]. C2C12 cells treated with LiCl (20 mM) were used as a positive control and FOP-flash reporter activity as a negative control. No differences in TCF reporter activity were detected when C2C12 cells were grown alone or co-cultured with PC3 Hem or PC3 control cells (Figure 6 B).

### Bone explant co-culturing with PC3 cells induces HO-1 expression

**Figure 7:**
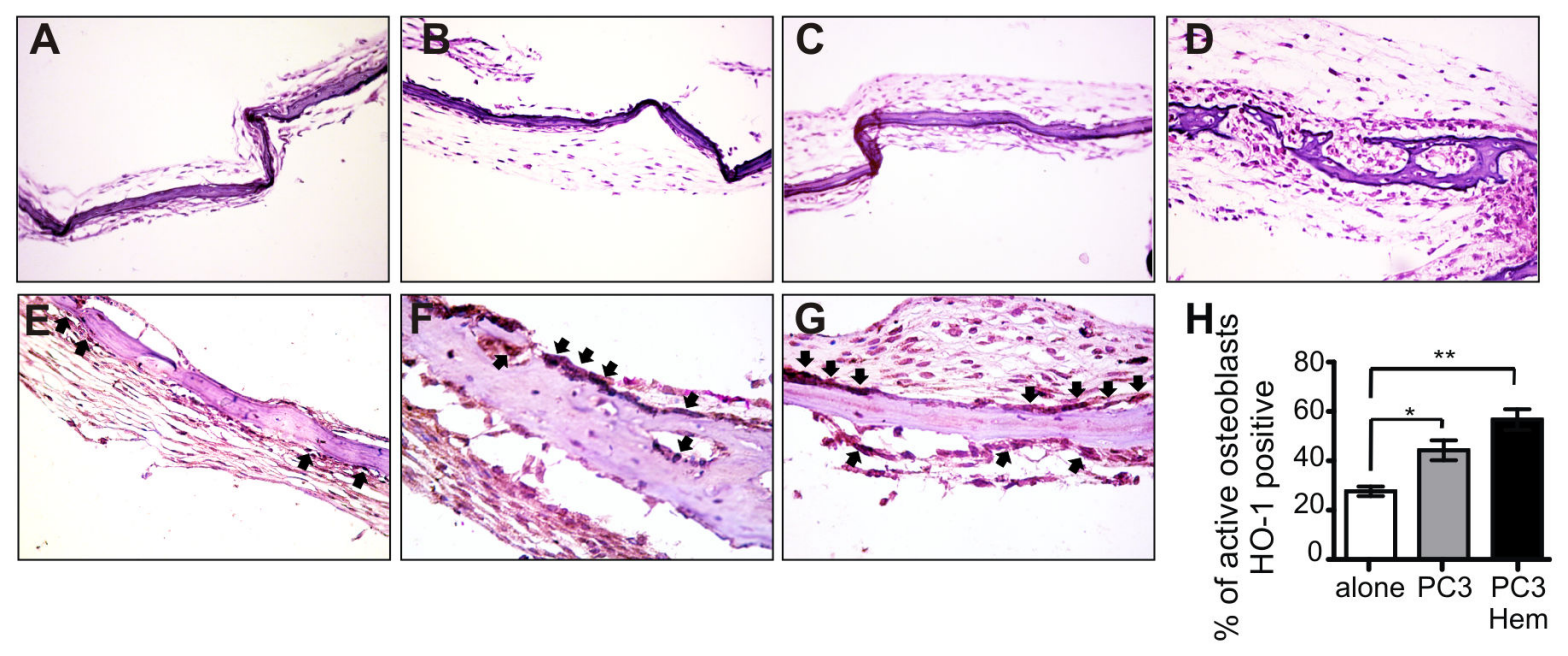
Co-culture with HO-1-induced PC3 cells increased HO-1 expression in bone explants. **Upper panel**. H&E staining of representative neonatal mouse calvariae cultured *in* *vitro* in the absence (**A**) or presence of PC3 cells control (**B**) or PC3 cells pre-treated with hemin (**C**). Insulin was used as a positive control of bone formation (**D**). **Lower panel**. Immunohistochemical staining of HO-1 of representative neonatal mouse calvariae cultured *in* *vitro* in the absence (**E**) or presence of PC3 cells control (**F**) or PC3 cells pre-treated with hemin (**G**). The arrows indicate the positive immunostaining in active osteoblasts. Magnification, x 250. The bands indicate the percentage of HO-1 positive osteoblasts respect to total active osteoblasts (**H**) (Significant difference, * *P*<0.05; ** *P*<0.01).

To extend these results, we used a bone organ culture assay. PC3 Hem or PC3 control cells were co-cultured with mouse calvaria explants. The histological analysis showed no differences in bone formation when calvarias were grown alone (control) or in co-culture (Figure 7 A-C). Using the immunohistochemistry technique we next explored the expression of HO-1. As shown in Figure 7 (E-H) the percentage of active osteoblasts with HO-1 positive reactivity was higher in the calvarias co-cultured with the tumor cells than in controls. This increase was even higher when the calvarias were co-cultured with PC3 Hem cells (Figure 7 H). Here we demonstrate that it is feasible to induce HO-1 expression in calvarias co-cultured with prostate tumor cells, probably as a defense mechanism to maintain the organ homeostasis.

### Growth of PC3HO-1 tumors in bone

**Figure 8:**
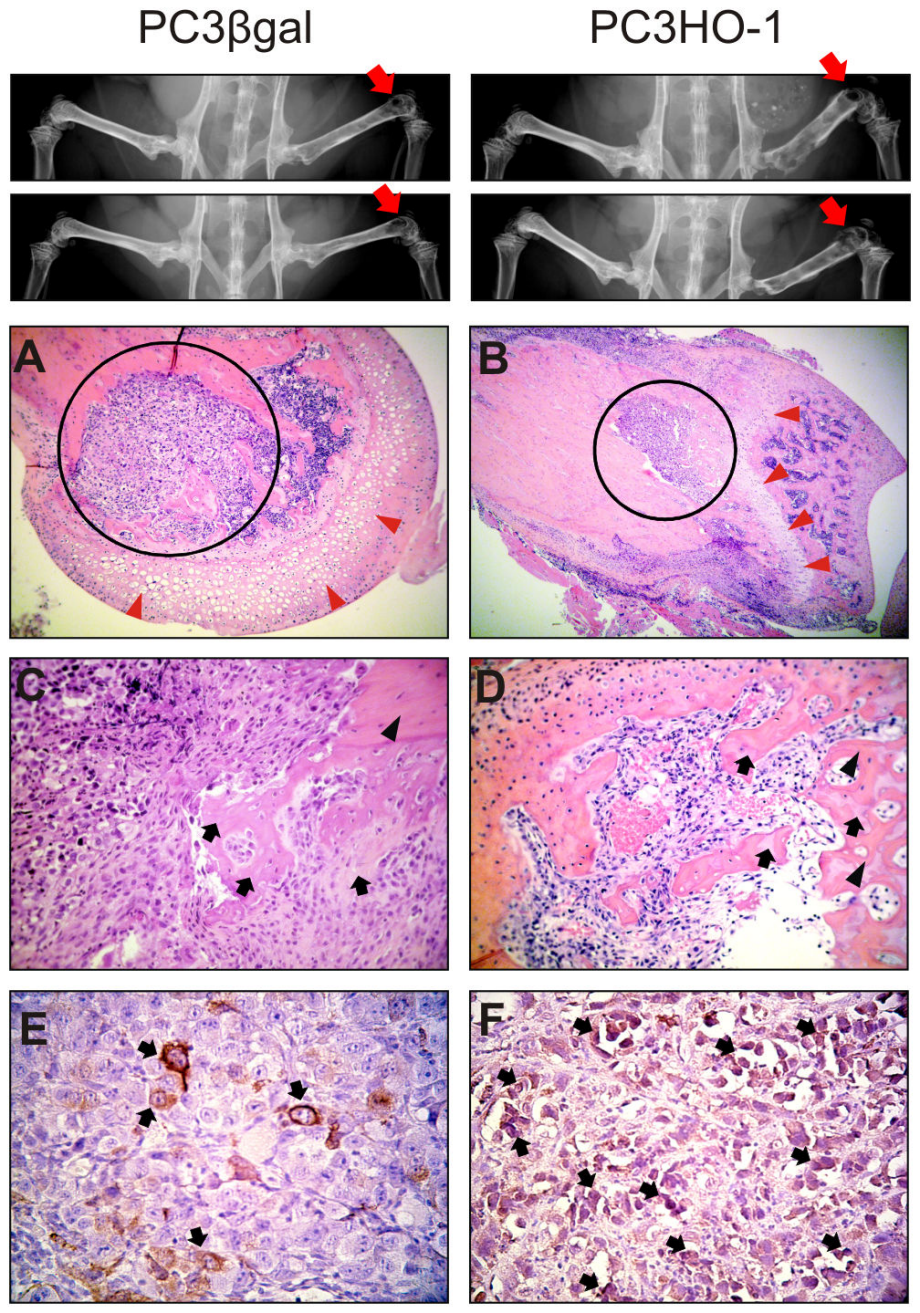
Histological analysis of intra-osseous metastasis. PC3HO-1 cells and PC3βgal were injected into distal head of the right femur of SCID mice. Upper panel, X-ray imaging of mouse legs after intrabone injection of PC3HO-1 or PC3βgal cells. Red arrow, injected limb. Lower panel, longitudinal section of mouse femur stained with H&E (A&B, magnification x 100; C, magnification x 400; D, magnification x 250). The red arrow heads indicate cartilage; black circle, tumor; black arrows, immature bone and black head arrows mature bone. Immunohistochemistry of HO-1 (E&F, magnification x 400) with scattered cells positive in cytoplasm (black arrows).

Because PC3 cells produce an osteolytic reaction, we next investigated the effect of HO-1 induction in PCa cells growing in the bone. We injected HO-1 stable transfected PC3 cells (PC3HO-1) or their controls (PC3βgal) in the femur of SCID mice. After 2 weeks, PC3HO-1 and PC3βgal derived-tumors were detected by X-rays and bone remodeling was observed. The bones injected with PC3HO-1 presented thickness and loss of the normal structure. Robust osteolytic reaction was detected in the PC3HO-1 bearing bones, with decalcified sites (Figure 8). None of the contralateral legs of any injected mouse showed evidence of bone lesions (Figure 8, X-rays). These findings were confirmed by histologic analyses of the samples, demonstrating the osteoblastic reaction by the presence of immature bone, mainly in PC3HO-1 metastasis (Figure 8). Furthermore, in tumors of both groups, mitotic figures of normal and abnormal features were evident. 

The forced expression of HO-1 in PC3 cells growing in the femur of SCID mice was confirmed by immunohistochemistry (Figure 8 E&F). Notably, HO-1 nuclear localization was observed in PC3HO-1 metastasis giving support to our previous reports revealing HO-1 expression in the nucleus of human primary prostate carcinomas [9], in PCa cell lines and in PC3HO-1 *s.c.* xenografts [10,12].

## Discussion

In this manuscript we have explored the effect of the induction of HO-1 in the interaction between PC3 cells and bone, the main site of this disease progression. Our studies indicate that hemin pre-treatment of PC3 cells restored osteoblasts proliferation probably by a mechanism mediated by FoxO activation. Furthermore, the inoculation of PC3HO-1 cells in the femur of mice produced strong bone remodeling. 

The pathogenesis of PCa reflects hereditary and environmental components. The cytokines, chemokines and MMPs are a proinflammatory network that contributes directly to the malignant progression and correlates with invasion, angiogenesis and metastasis [25].

We have previously suggested that HO-1 acts as a rheostat controlling prostate tumorigenesis. We demonstrated that HO-1 up-regulation induced its nuclear localization and inhibited cells proliferation, migration and invasion. Moreover, it impaired tumor growth *in vivo* and down-regulated the expression of target genes associated with inflammation and angiogenesis [10,11]. Furthermore, we recently documented that HO-1 antitumorigenic properties are partially due to the impairment of the androgen receptor signaling mediated by the blockade of the STAT3 axis [12]. Additionally, we demonstrated for the first time that HO-1 associates to promoters, such as uPA, PSA, MMP9, suggesting a novel function for this protein in the nucleus beyond its classic cytoplasmic enzymatic role in heme degradation [12]. 

In the bone microenvironment HO-1 mRNA has been shown to be present in osteoblast, osteocytes and osteoclasts [26]. However, the protein levels are very low in normal osteoblasts and osteoclasts.

Several factors released by PCa cells participate in the pathophysiology of prostate cancer progression in bone [27]. The prostate cancer cell line MDA PCa 2b, that produces mainly osteoblastic lesions, induced osteoblasts proliferation and differentiation and new bone formation *in vivo* while PC3 cells that produces an osteolytic reaction, inhibited osteoblasts proliferation *in vitro*. Furthermore, the WNT canonical pathway modulated the osteoblasts function and DKK1 was identified to be involved in the balance between bone formation and resorption determining the lesion phenotype [13,18]. In this paper we investigated the impact of the pharmacological induction of HO-1 in PC3 cells on bone response.

The imbalance in the oxidative status seems to participate in the pathogenesis of PCa and was suggested that the advanced disease is associated with a state of high oxidative stress [28]. Our results provide evidence that hemin treated-PC3 cells challenges the osteoblasts oxidative balance *in vitro*, reflected by the increased levels of the antioxidants enzymes. The presence of PC3 cells in the co-culture unveils a link between oxidative stress and the bone reaction. Their presence triggers the FoxO signaling cascade, partly responsible of the restoration of the proliferation of osteoblasts. It was proposed that the osteoblasts oxidative status may contribute to the establishment of the oxidative profile in PCa patients and the oxidative profile appears to be modified by bone metastasis, treatment and Gleason score [28]. Moreover, aggressiveness of PCa was demonstrated to be associated with a high oxidative stress status. In our *in vivo* experiments, the oxidative balance leads to bone remodeling and HO-1 induction in reactive osteoblasts. However, more studies are necessary to confirm the exact connection of the role of oxidative stress, HO-1 response and PCa-derived bone metastasis. 

In normal bone, there is a strict equilibrium between bone resorption and formation, including the maintenance of the bone metabolism and its structural integrity. Under inflammatory conditions the bone balance is interrupted, which depends on two critical factors RANKL and OPG [29]. In our study the presence of PC3 cells produced an increased in RANKL and a decreased in OPG, resulting in a net increase in the RANKL/OPG ratio and therefore in the bone resorption. It is clear that factors derived from PC3 cells impact bone balance [13]. In this work, we also observed that hemin pre-treatment of tumor cells altered PC3 milieu and in turn osteoblasts recovered their proliferative capacity, probably by re-gaining their homeostasis. The co-culture of PMOs with hemin-pretreated PC3 cells diminished the expression of CCL2, OPN and CSF-1, genes involved in osteoclastogenesis, migration, maturation and adhesion of osteoclasts. Furthermore, Runx-2 transcripts were significantly induced in PMOs by co-culturing with hemin-pretreated cells. Pratap et al [30] proposed Runx-2 as a bone microenvironment sensor that controls osteoblasts proliferation in response to growth signals. Thus, considering the regulatory role exerted by osteoblasts on osteoclasts, our data suggest that the co-culture of PMOs with HO-1-induced PC3 cells affected the dialogue between osteoblasts and osteoclasts. 

Inflammation is related to bone loss and inflammatory disorders are precipitators of enhanced bone resorption producing osteoporosis [31]. The onset of osteoclastogenesis and the consequent bone loss are precipitated by typical inflammatory molecules [32]. It was demonstrated that HO-1 influenced osteoclastogenesis *in vitro* and *in vivo*, that HO-1 activity negatively regulated inflammatory bone destruction and that HO-1 up-regulation impaired osteoclasts differentiation [26,33]. Vanella et al [34] and Bargaballo et al [35] reported that bone marrow mesenquimal stem cells displayed osteoblasts linage differentiation under HO-1 induction. This positive control exerted by HO-1 on osteoblasts differentiation was suggested to be associated to oxidative stress and to the induction of OPG and OCN [34]. Moreover, in osteoarthritis HO-1 was proposed to be an osteoblasts cytoprotective protein [36]. Lin et al [37], using primary cultured osteoblasts, demonstrated that HO-1 induction inhibited the maturation of osteoblasts including mineralized bone formation.

The results in the present study suggest that increased HO-1 expression in osteoblasts may be an adaptive mechanism to maintain the homeostasis of the bone tissue in response to the aggressiveness produced by the inflammatory milieu induced by PCa cells.

## Supporting Information

Figure S1 **HO-1 expression in PC3 cells co-cultured or not with PMOs.** PC3 cells were pre-treated with hemin (80 μM, 24 h, black columns) or not (control, white columns) and co-cultured or not with PMOs. HO-1 mRNA levels were determined by RT-qPCR (**A**) and protein levels by Western blot. Data were normalized to β-actin and were expressed as fold induction respect to PC3. One representative from at least three independent experiments is shown (Significant difference, * *P*<0.05).
(TIF)

Figure S2 **Von Kossa.** To determine calcified matrix deposition, von Kossa staining was applied to PMOs. After the co-culture, the PMOs grown alone (PMO), co-cultured with PC3 (PMO PC3) or with PC3 pre-treated with hemin (PMO PC3 Hem) were cultured for 21 days in differentiation medium and it was changed every 2 days.
(TIF)
