# Supplementary figures and images for: Heme Oxygenase-1 (HO-1) Expression in Prostate Cancer Cells Modulates the Oxidative Response in Bone Cells
Source: PLoS One. 2013 Nov 4;8(11):e80315. doi: 10.1371/journal.pone.0080315 (PMC3817116; doi:10.1371/journal.pone.0080315)

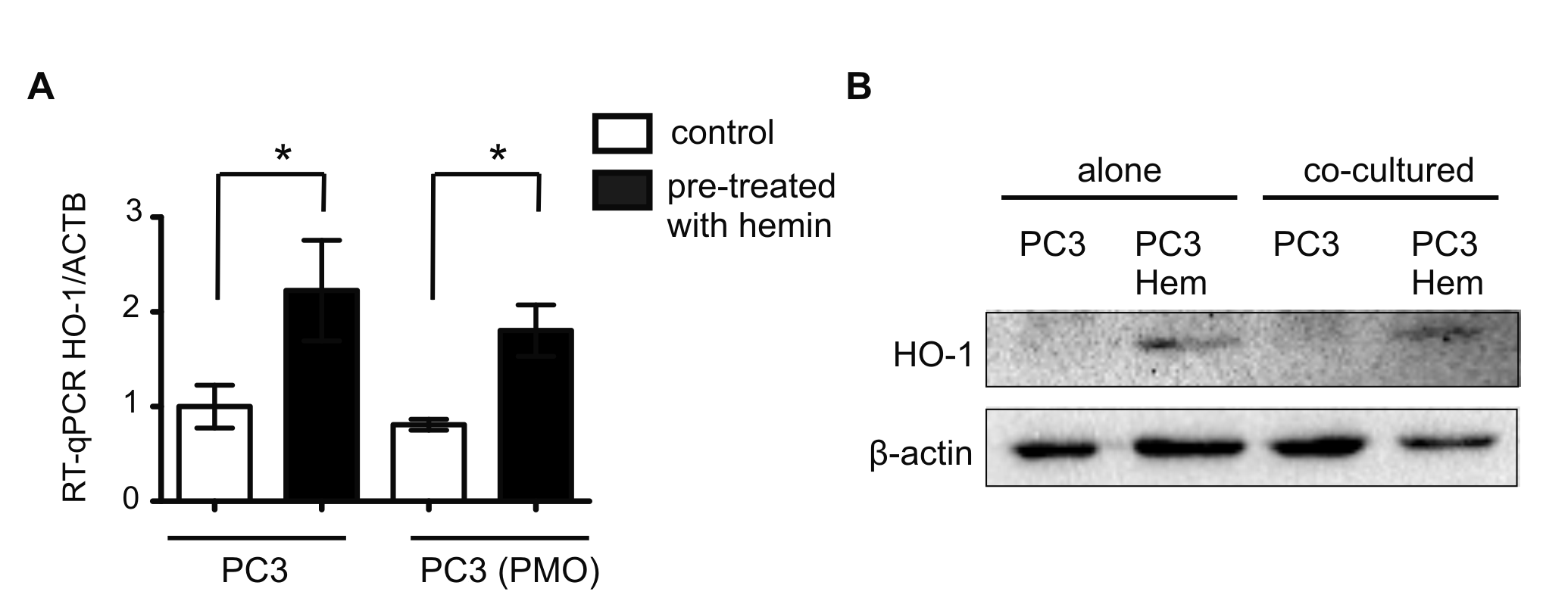

Supplement: Figure S1 — HO-1 expression in PC3 cells co-cultured or not with PMOs. PC3 cells were pre-treated with hemin (80 μM, 24 h, black columns) or not (control, white columns) and co-cultured or not with PMOs. HO-1 mRNA levels were determined by RT-qPCR (A) and protein levels by Western blot. Data were normalized to β-actin and were expressed as fold induction respect to PC3. One representative from at least three independent experiments is shown (Significant difference, * P<0.05). (TIF) [file pone.0080315.s001.tif]

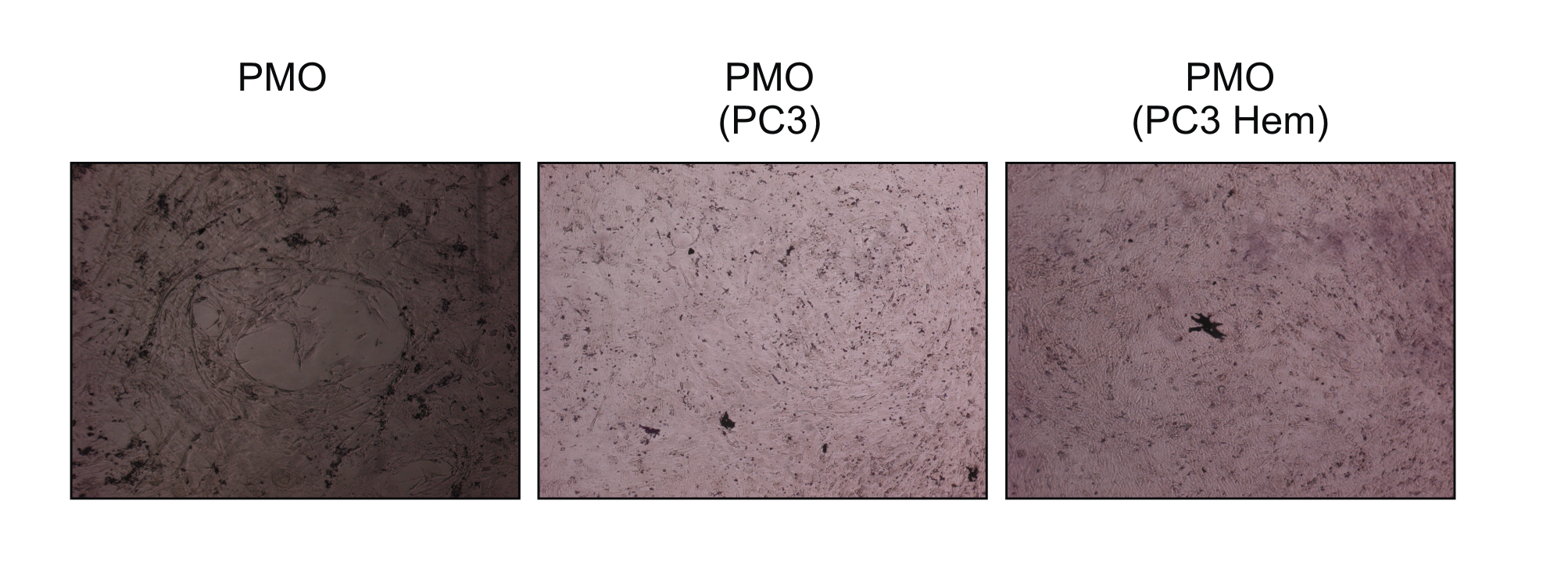

Supplement: Figure S2 — Von Kossa. To determine calcified matrix deposition, von Kossa staining was applied to PMOs. After the co-culture, the PMOs grown alone (PMO), co-cultured with PC3 (PMO PC3) or with PC3 pre-treated with hemin (PMO PC3 Hem) were cultured for 21 days in differentiation medium and it was changed every 2 days. (TIF) [file pone.0080315.s002.tif]
